# Sudden Death in Pleural Effusion Cases

**Published:** 1908-05-02

**Authors:** 


### Medicine.

### SUDDEN DEATH IN CASES OF PLEURAL EFFUSION.

It is well known that in a case of pleural effusion in which one side of the chest is so full of fluid that it is completely dull to percussion from top to bottom, there is a liability to sudden death. The question arises: Why should this be so? It is by understanding the reasons for it that steps can be adopted to avoid such a catastrophe.

At the autopsies that have been made upon cases of the kind definite causes for death have been found in a small number of instances; such causes, for example, as thrombosis of a pulmonary artery, pulmonary embolism, cerebral hæmorrhage, rupture of an empyema into a bronchus, myocardial degeneration, and so forth. In the great majority of the cases, however, there is nothing found at the autopsy to account for sudden death. The pleuritic effusion is there, but there is nothing macroscopic to account for the clinical fact that, whereas for days this same effusion was well borne, all in a moment something led to immediate death.

### Dangers of the Sitting Posture.

It seems very likely, therefore, that the immediate cause of death in these cases is a sudden alteration in the vascular conditions within the thorax. That this is really so is further suggested by the fact that this sudden death seldom or never occurs when the patient remains perfectly still, but only when there has been a sudden change of his position. The most dangerous of all such alterations in position is that from a recumbent to a sitting posture. In nearly every case in which a large pleural effusion has led to sudden death it has been when the patient has sat up in bed.

When the patient remains always in the same position the circulation of the blood within the venæ cavæ and auricles of the heart becomes accommodated to the external pressure to which the blood within these tubes and cavities is subjected.

### The Effects of Reduced Intrathoracic Pressure.

It is clear that if a large pleural effusion of five or six pints could be poured out within a few moments there would be no time for the pressure of the blood circulating within the intrathoracic veins to become accommodated to the suddenly increased pressure outside those veins, and the patient would be dead in a moment or two from stoppage of the circulation. Such sudden effusions into a pleural cavity, fortunately, do not occur; even in the acutest cases the rate of effusion is such that there is time for the intrathoracic venous circulation to adapt itself to the rapidly, but not suddenly, increasing pressure outside. Dyspnœa is common until such accommodation has become established, and then it disappears if the patient keeps still. The pressure within that pleural cavity into which the effusion has taken place will be greater or less according to the amount of fluid that the confined space is compelled to hold, and at any given time this general pressure of the pleural effusion will be constant, though it will vary gradually according as the activity of the inflammatory lesion increases or abates.

### The Factor of Gravity.

In addition, however, to this general intrapleural pressure, which is at any moment constant, there is the factor of gravity to be taken into account as well. The hydrostatic pressure caused by gravity will vary with the vertical height of the column of fluid that bears upon any given point, and it is greatest at the bottom of the cavity. If the patient be lying upon his back the height of the column of pleuritic fluid above the level of the inferior vena cava will be several inches less than when he sits up. If the circulation is only just maintained when the hydrostatic pressure upon the outside of the inferior cava is that of the procumbent position, it may when the patient sits up be obstructed altogether. Granted time, the pressure in the abdominal portion of the inferior vena cava will slowly rise until blood will be driven past even this obstruction; but it is just this time which the patient is unable to give; he is dead before the accommodation can be brought about. If the patient has been sitting up and then suddenly lies down there is a similar sudden disturbance of the distribution of the intrathoracic pressure, but it is less severe in degree than when the patient passes from the recumbent to the sitting posture.

### Some Rules to be Observed.

The thing to be borne in mind is that it is not so much the actual pressure of the pleural effusion as the suddenness of any change in its distribution that is dangerous. The practical points that can be deduced from this are:—

1. That a patient of the kind should on no account be allowed to sit up or change his position suddenly.
2. That such a patient should consequently always be dealt with at his own home; he should on no account be transferred to a hospital or elsewhere until steps have been taken to lessen the quantity of fluid in his chest.
3. That the gradual removal of the fluid through a Southey's tube is infinitely preferable to its sudden release by aspiration.
4. That the very greatest care should be taken not to move the patient about unduly, either for purposes of physical examination of the chest or for cleansing the skin before the insertion of the Southey's tube. It is so much easier to wash the chest wall and insert the tube when the patient is in the sitting position that unless particular instructions are given to the nurse the patient is very apt to be allowed to sit up before the doctor can stop it.

The general condition of the patient is no guide as to whether sitting up may be allowed or not. He may say he is perfectly able to sit up, and in his vigour he may move with such energy that a minute or two later he is dead. When a pleural cavity is quite full of fluid all sudden movement of every kind should be absolutely avoided until part at least of the fluid has been removed.

